# A New Look for the Red Macroalga *Palmaria palmata*: A Seafood with Polar Lipids Rich in EPA and with Antioxidant Properties

**DOI:** 10.3390/md17090533

**Published:** 2019-09-13

**Authors:** Diana Lopes, Tânia Melo, Joana Meneses, Maria H. Abreu, Rui Pereira, Pedro Domingues, Ana I. Lillebø, Ricardo Calado, M. Rosário Domingues

**Affiliations:** 1 Centro de Espectrometria de Massa, Departamento de Química & QOPNA-LAQV, Universidade de Aveiro, Campus Universitário de Santiago, 3810-193 Aveiro, Portugal; 2 Departamento de Química & CESAM & ECOMARE, Universidade de Aveiro, Campus Universitário de Santiago, 3810-193 Aveiro, Portugal; 3 ALGAplus—Produção e Comercialização de algas e seus derivados, Lda., 3830-352 Ílhavo, Portugal; 4 Departamento de Biologia & CESAM & ECOMARE, Universidade de Aveiro, Campus Universitário de Santiago, 3810-193 Aveiro, Portugal

**Keywords:** IMTA, lipidome, lipidomics, mass spectrometry, rhodophyta, seaweeds

## Abstract

*Palmaria palmata* is an edible red macroalga widely used for human consumption and valued for its high protein value. Despite its low total lipid content, it is rich in eicosapentaenoic acid (EPA). This seaweed has been scarcely explored with regard to its lipid composition. The polar lipids of seaweeds are nowadays recognized as important phytochemicals contributing to their add value valorization and providing support for claims of potential health benefits. The present study aimed to disclose the polar lipid profile of *P. palmata*, farmed in an integrated multi-trophic aquaculture (IMTA) through modern lipidomic approaches using high-resolution LC-MS and MS/MS and to screen for the antioxidant properties of this red macroalga. A total of 143 molecular species of lipids were identified, belonging to several classes of polar lipids, such as glycolipids, phospholipids, and betaine lipids. It is noteworthy that the most abundant lipid species in each class were esterified with eicosapentaenoic acid (EPA), accounting for more than 50% of the lipid content. The polar lipid extract rich in EPA showed antioxidant activity with an inhibition concentration (IC) of IC30 = 171 ± 19.8 µg/mL for α,α-diphenyl-β-picrylhydrazyl radical (DPPH^●^) and IC50 = 26.2 ± 0.1 µg/mL for 2,20-azino-bis-3-ethylbenzothiazoline-6-sulfonic acid radical cation (ABTS^●+^). Overall, this study highlights that *P. palmata* farmed in an IMTA framework can be a sustainable source of beneficial lipids with antioxidant activity. Moreover, this red macroalga can be exploited for future applications as a source of lipids rich in EPA for food and feed, nutraceuticals, and cosmetics.

## 1. Introduction

Over the last 20 years, macroalgae have ceased to be a trifle in western countries and have been considered as a nutritional and functional food, generating wealth for many coastal countries [1]. Red macroalgae (phylum Rhodophyta) are mainly used as a source of hydrocolloids (agar and carrageenans), generating a large turnover worldwide [2]. Nevertheless, the marketing of edible red macroalgae is also very important, as is the trade of Asian species of *Porphyra* sp., the most valued variety of seaweed in the world, generally recognized by its common Japanese name, nori. Recently, some of the species of the genus *Porphyra* were transferred to the genus Pyropia [3]. Food and agriculture organization of the united nations (FAO) data indicate that the production of *Porphyra* sp. is global, but Japan, China, and the Republic of Korea are still the main producers [4]. From 2000 to 2016, the Global Aquaculture Production for *Porphyra* sp. increased from 424,913 to 1,352,520 tons, demonstrating the enormous demand for these edible red seaweeds [4]. Nonetheless, there is another valuable red macroalga that has remained largely overlooked, *Palmaria palmata* ((Linnaeus) F. Weber & D. Mohr, 1805), also known as dulse (Palmariales, Rhodophyta). It is an edible red macroalga found in the coastal waters of the North Atlantic [5]. Its lipid content, as in most macroalgae, compared to other photosynthetic lipid sources is considered low (0.3–3.8% of dry weight) and erroneously devalued [6]. However, the lipids of *P. palmata* are easily assimilated by the human body and have a high content of eicosapentaenoic acid (EPA), a golden fatty acid (FA) in healthy diets and important in the prevention of non-communicable diseases (NCDs), which are responsible for the deaths of 41 million people each year (equivalent to 71% of all deaths worldwide) [7,8]. Eicosapentaenoic acid is an omega-3 (*n*-3) polyunsaturated fatty acid(PUFA) that plays an effective role in the improvement and prevention of cardiovascular and neurodegenerative diseases, as well as beneficial antioxidant and anti-inflammatory effects [9,10,11]. The PUFA of seaweeds are generally located in the structural lipids found in their membranes in the form of phospholipids (PLs) and glycolipids (GLs). To our best knowledge, the polar lipids of *Palmaria palmata* have not been studied so far, and the lack of knowledge on this subject must be overcome because polar lipids bearing EPA have recently been associated with several bioactive properties, such as antitumor [12,13], anti-inflammatory [14,15], antimicrobial [16,17], and antiviral [18]. Thus, the identification of the *P. palmata* lipidome is crucial for future exploitation and valorization. In addition, seaweed lipids are also recognized as natural and organic antioxidants with different applications [19]. Today, there is an increasing demand for new antioxidants for the food, feed, nutraceuticals, and cosmetic industry, and consequently, an increasing bioprospecting effort. Food containing phytochemical antioxidants can be used as nutritional supplements and functional foods and to extend the shelf life of food for human consumption. In addition, the cosmetic industry is increasingly looking for natural antioxidants recognized as active ingredients for cosmetic formulations that alter the effects of ageing and act as inhibitors of oxidants [20]. Nevertheless, to our best knowledge, the lipid antioxidant potential of *P. palmata* has not yet been explored. 

Knowing the lipid profile of macroalgae can help to reveal their healthy attributes and their added value. The use of lipidomics approaches based on liquid chromatography mass spectrometry (LC-MS) has been successfully used for this purpose [21,22,23,24,25] but has not yet been applied to the profiling of *P. palmata*. The present study characterized the polar lipid signature of *P. palmata* farmed in an integrated multi-trophic aquaculture (IMTA) system. A lipidomic approach based on LC-MS was used to detect lipids bearing *n*-3 fatty acids. Also, the antioxidant activity of the polar lipid-rich extract was also screened, through the free radical scavenging potential against α,α-diphenyl-β-picrylhydrazyl (DPPH) and 2,20-azino-bis-3-ethylbenzothiazoline-6-sulfonic acid (ABTS) radicals. Our goal was to add value to *P. palmata* by presenting it as a bioactive lipid source and EPA carrier with potential applications in the fields of food, feed, pharma, and cosmetics.

## 2. Results

### 2.1. Identification of the of Palmaria Palmata Lipidome 

The lipid extracts of *P. palmata* represented an average yield of 1% by dry weight (255.6 ± 1.19 mg of biomass, 2.58 ± 0.19 mg lipid extract). The fatty acid composition of the lipid extract of *P. palmata*, identified by gas chromatography-mass spectrometry (GC-MS) (Table 1), shows the prevalence of 20:5(*n*-3) EPA with a relative abundance of 51.68% ± 6.5. The saturated FA 16:0, 18:0, and 14:0 represented 24.32% ± 1.11, 12.45% ± 6.74, and 5.32 ± 0.44, respectively. The others FA identified had relative abundances of less than 5%. The GC-MS analysis also identified phytol compounds, a component of chlorophyll and vitamin E and K. Phytol is a precursor of vitamin E, an important nutritional supplement obtained commercially by isolation from natural sources [26].

**Figure 1:**
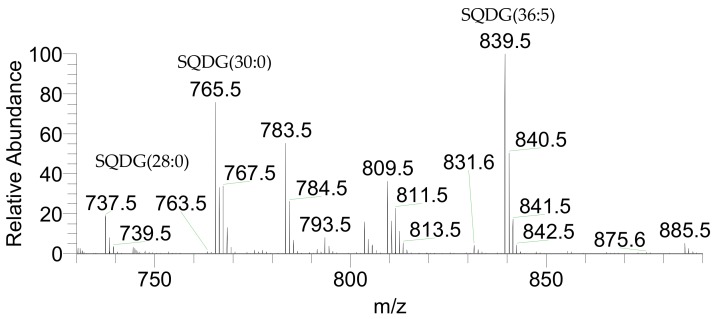
LC-MS spectrum representative of the class SQDG *of P. palmata* identified as [M − H]^−^ ions.

**Figure 2:**
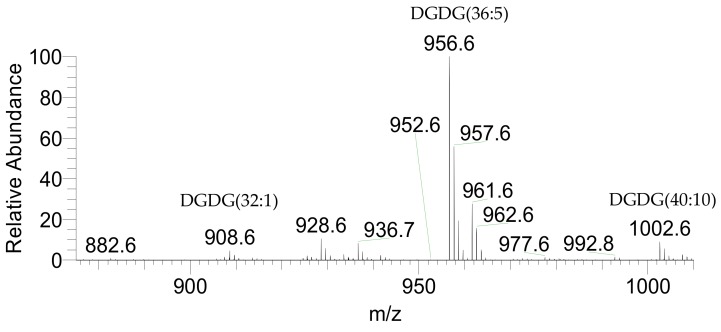
LC-MS spectrum representative of *P. palmata* DGDG class identified as [M + NH_4_]^+^ ions.

Identification of the polar lipid profile at the molecular level was performed by high-resolution hydrophilic interaction liquid chromatography-mass spectrometry - HILIC-LC-MS and HILIC-LC-MS/MS. In total, 46 molecular species of glycolipids were identified, 1 molecular species of betaine lipids, 91 molecular species of phospholipids, and 6 molecular species of inositephosphoceramide lipids, representing a total of 144 species of lipids. The glycolipids identified included the acidic glycolipids sulfoquinovosyl diacylglycerol (SQDG) (Figure 1) and sulfoquinovosyl monoacylglycerol (SQMG) classes, assigned as [M − H]^−^ ions in the LC-MS spectra (Table 2) and the neutral glycolipids monogalactosyldiacylglycerol (MGDG), monogalactosylmonoacylglycerol (MGMG), digalactosyldiacylglycerol (DGDG) (Figure 2) plus digalactosylmonoacylglycerol (DGMG) classes, identified in the positive LC-MS spectra as [M + NH_4_]^+^ ions (Table 3). The most abundant species in each class of glycolipids was assigned as SQDG (36:5) corresponding to SQDG (20:5/16:0), SQMG (14:0), DGDG (36:5) as DGDG (20:5/16:0), DGMG(16:0), MGDG (40:10) as MGDG (20:5/20:5), and MGMG (20:5).

Betaine lipids of *P. palmata* included only one species of diacylglyceroltrimethylhomoserine (DGTS) assigned as DGTS (32:5), with a molecular formula of C_42_H_80_O_7_N, and identified in the LC-MS spectra as positive [M + H]^+^ ions. Its theoretical *m/z* is 710.5935 and the observed *m/z* was 710.5936 (0.14 ppm).

**Figure 3:**
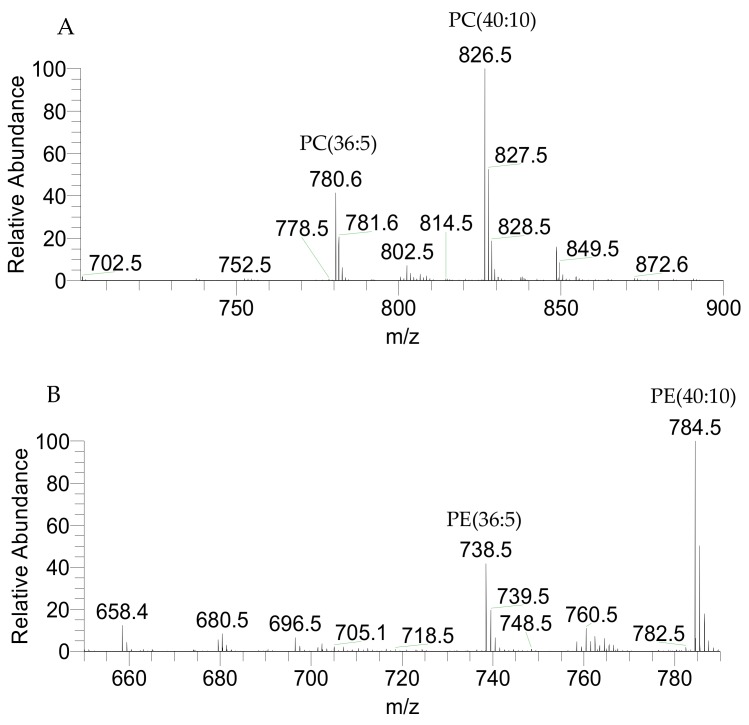
LC-MS representative spectra of *P. palmata* PC (**A**) and PE (**B**) classes identified as [M + H]^+^ ions.

**Figure 4:**
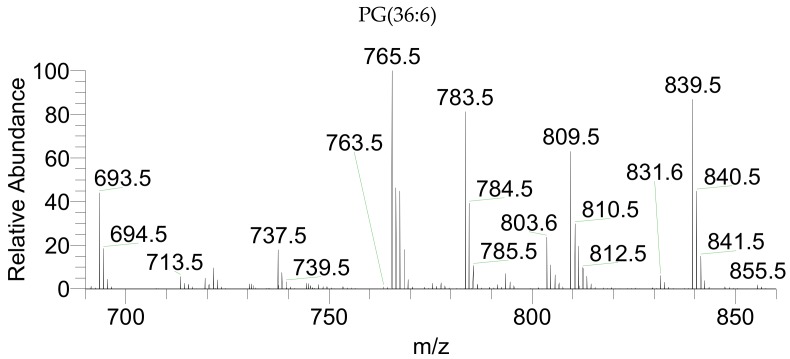
LC-MS representative spectrum of *P. palmata* PG class identified as [M − H]^−^ ions.

The classes of phospholipids found in *P. palmata* included phosphatidylcholine (PC) (Figure 3A), lyso-PC (LPC), phosphatilylethanolanime (PE) (Figure 3B), and lyso-PE (LPE) were identified in the LC-MS spectra as positive [M + H]^+^ ions (Table 4). Phosphatidylglycerol (PG) (Figure 4), lyso-PG (LPG), phosphatidylinositol (PI), and phosphatidic acid (PA) were identified in the LC-MS spectra as negative [M − H]^−^ ions (Table 5). Also, inositephosphoceramide lipids (IPC) were identified as negative [M + CH_3_COO]^−^ ions (Table 6). The PC(40:10) identified as PC(20:5/20:5), LPC(20:5), PE(40:10) identified as PE(20:5/20:5), LPE(20:5), PG(36:6) as PG(16:1/20:5), LPG(14:0), PI(40:10) as PI(20:5/20:5), PA(40:10) as PA(20:5/20:5), and PI-Cer(d40:2) represent the most abundant species within each phospholipid class.

**Figure 5:**
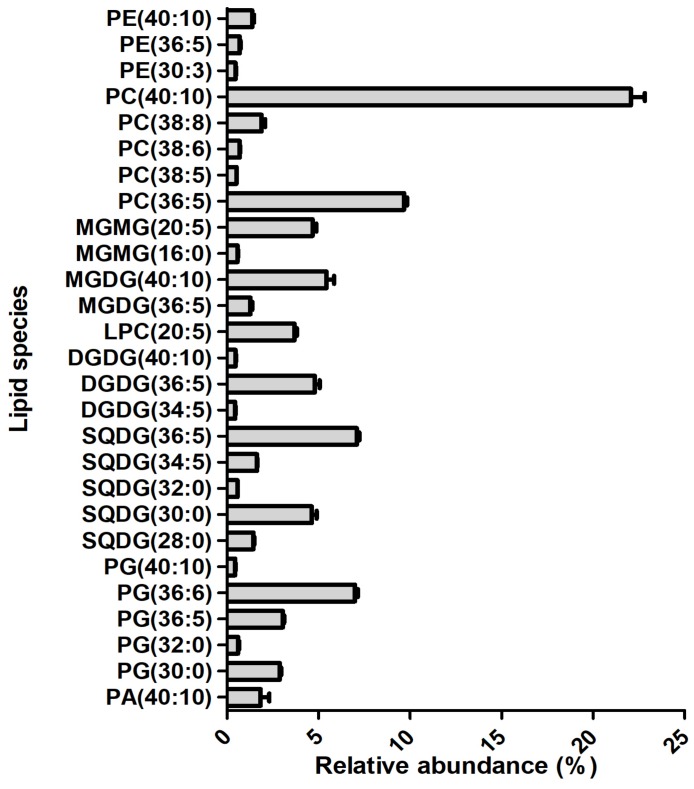
The relative percentage of lipid contents equal to or greater than 0.5% of *P. palmata*. Values are averages of five samples ± standard deviation.

**Figure 6:**
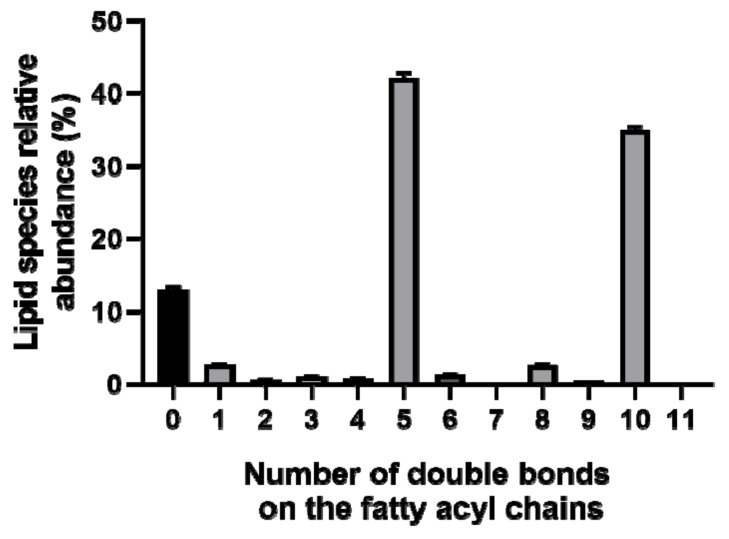
The relative abundance of lipid species of *P. palmata* including the same number of double bonds on fatty acyl chains.

The relative quantification of the identified species was calculated after integrating the peak area of each identified ion, normalized to the internal standard and divided by the sum of the normalized area of all species. Lipid species identified with a relative percentage of 0.5% or greater are shown in Figure 5. Phospholipids were the group of lipids with the largest number of species (91 species). Particular emphasis is placed on PC(40:10), assigned as PC(20:5/20:5), which was the most abundant identified lipid species, with an average relative percentage of 23.7%. The PC(36:5), assigned as PC(16:0/20:5) was the second most abundant species, with an average relative percentage of 10.4%. It remains important to highlight the lipid species SQDG(36:5), MGDG(40:10), DGDG(36:5), SQDG(30:0), and MGMG(20:5), with an average relative percentage of 5% or greater. Both PC species are EPA carriers. The 20:5 fatty acyl chain was confirmed by LC-MS/MS spectra data analysis of the above-mentioned species, with the exception of SQDG (30:0). Indeed, the lipid molecular species with 5 and 10 double bonds on the fatty acid acyl chains are clearly pronounced (Figure 6), corroborating the prevalence of EPA. 

### 2.2. Evaluation of Antioxidant Activity 

**Figure 7:**
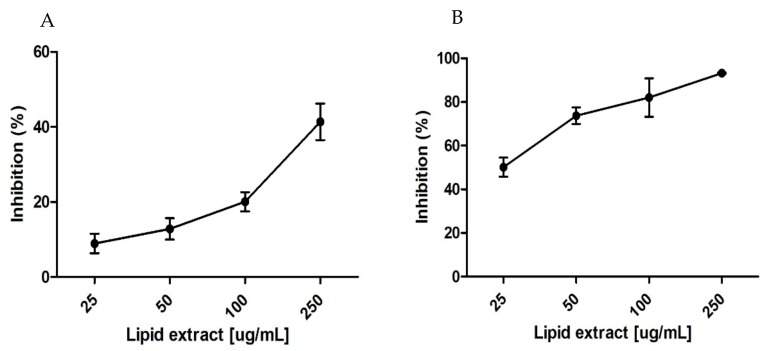
Percent inhibition as a function of the concentration of the lipid extract after 120 minutes of DPPH^●^ (**A**) and ABTS^●+^ (**B**) radical scavenging activity.

The antioxidant potential of *P. palmata* polar lipid-rich extract was evaluated through free radical DPPH^●^ and ABTS^●+^ scavenging assays. While DPPH assay measures the ability of antioxidants to scavenge the DPPH^●^ generated in the organic phase, the ABTS^●+^ assay acts in the same way in the aqueous phase. The percentage of radical inhibition in the presence of extracts rich in polar lipids was calculated after 120 minutes. The percent inhibition is proportional to time and the lipid extract concentration, even though the response appeared to be non-linear, with better performance for the ABTS^●+^ assay (Figure 7). 

Results gathered in these assays showed that for the DPPH assay, the polar lipid-rich extract concentration providing 30% of inhibition (IC30) was 171 ± 19.8 µg/mL with a TE of 88.7 ± 7.7 Trolox µmol/g lipid. The polar lipid-rich extract concentration providing 50% inhibition (IC50) in the ABTS assay was 26.2 ± 0.1 µg/mL with a TE of 555.4 ± 28.1 Trolox µmol/g lipid. The reactivity and the capacity of the extract rich in polar lipids to scavenge these radical were very different. While for the ABTS assay it was possible to calculate 50% inhibition, in the case of the DPPH assay, the maximum percentage of inhibition reached nearly 30%. 

## 3. Discussion

The consumption of long-chain PUFA, particularly *n*-3, may have long-term health benefits. Their positive effects include reduced cardiovascular disease, morbidity, and mortality [30,31,32]; enhanced visual and neurological development; and improved of inflammatory conditions, such as arthritis and asthma [33,34,35]. It is recognized that seafood is the main source of *n*-3 long-chain PUFAs, mainly EPA and docosahexaenoic acid (DHA). However, global overexploitation and depletion of marine fish stocks is of long-standing concern, as existing populations of wild and farmed species are unlikely sufficient to meet *n*-3 PUFA requirements for human consumption. In this context, *P. palmata* appears as an interesting source of EPA that can be supplied in a socio-ecologically sustainable way. Namely its production in IMTA systems compared to current wild harvesting practices might ensure the availability of biomass with increased stability of its nutritional value and biochemical profile, namely its polar lipids and its PUFA composition [36,37]. 

As described above (Table 1, Table 2, Table 3 and Table 4), PL and GL of *P. palmata* are quite rich in EPA. Our work is consistent with previous studies that reported high levels of EPA in *P. palmata* compared to other edible seaweeds [8,38].

Phospholipids, in particular those rich in PUFA (namely EPA), contribute significantly to the added value of *P. palmata*. There is evidence that PLs are better delivers of PUFA than triglycerides (TGs) [39,40]. Several studies suggested that much of the dietary PLs fraction is integrated into high-density lipoprotein (HDL) in the intestine, later joining the plasma HDL pool [41,42,43]. Since PLs are associated with low toxicity, allowing their use for any route of administration, PL from *P. palmata* are potential candidates for food and nutraceutical formulations.

Phosphatidylcholine is the most abundant phospholipid class of *P. palmata*. Administration of PCs has been reported to be beneficial for senescence, cognitive function, inflammatory diseases, and plasma and hepatic lipid metabolism [44,45,46,47]. It can also be used as a choline supplier. Choline is a component of the vitamin B complex, essential for human nutrition and used by the body to produce acetylcholine, one of the major neurotransmitters of the nervous system, involved in neural networks associated with memory [48]. Choline supplementation is important for vegans and vegetarians, who are at a higher risk for choline deficiency. Some studies also linked high choline intake with reduced risk of breast and colorectal cancer [49,50,51]. Thus *P. palmata* PLs could be considered as a potential functional food or as an ingredient for the fortification of foods for nutraceutical applications.

Other potential uses of *P. palmata* PLs are either in the cosmetics or in the pharma industries. Cosmetic formulations use the emulsifying properties of PLs for skin moisturizing products [52]. Pharmaceutical applications of PLs rely on their ability to form liposomes after mixing in aqueous media, making them a potential drug delivery vector [52,53]. Therefore, *P. palmata* PLs can potentially be used as emulsifiers, as an *n*-3 FAs supplement, and as beneficial nutritional biomolecules. Macroalgae are also considered a promising source of value-added phytochemicals. Several studies have reported that glycolipids can also display bioactive activities, including antioxidant [54], anti-inflammatory [14], anti-proliferative [55], and anti-microbial [56] activities. Some of the glycolipids identified in *P. palmata* have already been described as having bioactive properties, such as SQDG(34:2) with antiviral activity against HCM-virus [16], SQMD(16:0) with antibacterial activity against *Xanthomonas oryzae* [57], and the SQDG carrier of EPA with anti-proliferative effect on the inhibition of human telomerase [12] and DNA polymerase α and β [58]. Additionally, a study by Banskota and collaborators [14] attributed the anti-inflammatory action of *P. palmata* to polar lipids, including SQDG(20:5;14:0), via the inhibitory activity of nitric oxide, highlighting the role of EPA. Moreover, a MGDG carrier of EPA was patented because of its anti-inflammatory action [59]. The search for natural antioxidants has long attracted the attention of researchers, since inflammation is associated with oxidative stress occurring in several diseases, including chronic and age-related ones [60,61]. Oxidative stress occurs when the production of radicals increases and exceeds the detoxification capacity of cellular antioxidant defense systems. Antioxidants are biomolecules that prevent, or delay damage to other molecules, by the presence of free radicals, because they have the ability to block or inhibit them directly or indirectly, or because they stimulate cellular antioxidant defenses [62]. Antioxidants from natural or organic sources aim not only to promote the development of new drugs but also to act in the prevention of inflammatory diseases [63]. Such biomolecules with antioxidant properties can be obtained from foods, including plants and marine vegetables [64,65]. There are some studies of seaweed lipid characterization using lipidomics based on the LC-MS approach that allowed the putative bioactivity and health benefits of seaweeds lipids to be highlighted, as well the exploitation of seaweeds as a functional food, as reported for *Codium tomentosum*, *Porphyra dioica*, *Gracilaria* sp, *Chondrus crispus*, *Ulva rigida*, *Saccharina latissima*, and *Fucus vesiculosus* [21,22,23,24,25,66,67]. However, seaweeds’ lipids antioxidant potential was not explored.

The antioxidant activity of seaweeds has been mostly related to their phenolic compounds and pigment contents [68,69,70,71,72], whereas it is only now that lipids are beginning to deserve special attention. To the best of our knowledge, there is only one study of lipids’ fraction antioxidant activity for the red and brown seaweeds *Solieria chordalis* ((C.Agardh) J. Agardh, 1842) and *Sargassum muticum* ((Yendo) Fensholt, 1955), respectively, whose antioxidant potential was lower than the one obtained in this study for *P. palmata* [19]. Overall, our results showed that the polar lipid-rich extract of *P. palmata* exhibits antioxidant proprieties, showing a higher percentage inhibition of DPPH and TE than those of the methanolic extract *of P. palmata*, mainly composed of phenolic compounds, as previously described [68]. It also showed significantly better antioxidant activity for the DPPH^●^ and ABTS^●+^ assays compared to the red seaweed *Gracilaria manilaensis* (Yamamoto & Trono, 1994) organic extract [73]. The polar lipid-rich extract of *P. palmata* still had a higher antioxidant potential for the ABTS^●+^ assay compared to a study of antioxidant activity using different organic solvents with phenolic content from the brown seaweed *Sargassum serratifolium* ((C. Agardh) C. Agardh, 1820) [74]. 

## 4. Materials and Methods 

### 4.1. Reagents

Phospholipid standards 1,2-dimyristoyl-*sn*-glycero-3-phosphocholine (dMPC), 1,2-dimyristoyl-*sn*-glycero-3-phosphoethanolamine (dMPE), 1,2-dimyristoyl-*sn*-glycero-3-phospho-(1′-rac-)glycerol (dMPG), 1,2-dimyristoyl-*sn*-glycero-3-phospho-L-serine (dMPS), 1′,3′-bis[1–dimyristoyl-*sn*-glycero-3-phospho]-glycerol (tMCL), 1,2-dipalmitoyl-*sn*-glycero-3-phosphatidylinositol (dPPI), *N*-palmitoyl-D-erythro-sphingosylphosphorylcholine (NPSM), and 1-nonadecanoyl-2-hydroxy-*sn*-glycero-3-phosphocholine (LPC) were purchased from Avanti Polar Lipids, Inc. (Alabaster, AL). Chloroform (CHCl_3_), methanol (MeOH), ethanol absolute, and acetonitrile were purchased from Fisher scientific (Leicestershire, UK); all the solvents were of high-performance liquid chromatography (HPLC) grade and were used without further purification. DPPH^●^ was purchased from Aldrich (Milwaukee, WI). 2,20-Azino-bis(3-ethylbenzothiazoline-6-sulfonic acid) diammonium salt (ABTS^●+^) was obtained from Fluka (Buchs, Switzerland). Ammonium acetate and 6-hydroxy-2,5,7,8-tetramethylchromane-2-carboxylic acid (Trolox) were purchased from Sigma-Aldrich (St Louis, MO, USA). All the other reagents and chemicals used were of the highest grade of purity commercially available. Milli-Q water was also used (Synergysup^®^, Millipore Corporation, Billerica, MA, USA).

### 4.2. Seaweed Biomass

The biomass *of Palmaria palmata* ((Linnaeus) F.Weber & D.Mohr, 1805) was provided by a local aquaculture producer-ALGAplus-who farms seaweed integrated with finfish production (located at Ria de Aveiro coastal lagoon, mainland Portugal, 40°36′43″N, 8°40′43″W). *Palmaria palmata* is produced by vegetative propagation, in a controlled indoor system from June to September (approximately) and in an open-flow outdoor tank system during colder months. For this work, the red algae were harvested in May 2018, cleaned to remove the epiphytes, and washed using sterilized seawater. It was then dried at 25 °C in an air tunnel until 10% to 12% total moisture was reached. Five 250-mg aliquots were obtained from bulk production and were used for total lipid extraction. 

### 4.3. Lipid Extraction

The biomass of *P. palmata* was cut into small pieces and ground in a mortar and pestle with liquid nitrogen until homogenized. The lipid extraction procedure was performed using a modified Bligh and Dyer protocol [23], mixing 250 mg of seaweed biomass (five replicates) with 2.5 mL of MeOH and 1.25 mL of CHCl_3_ in a glass PYREX tube and homogenized by vortexing for 2 min and incubation in ice on a rocking platform shaker (Stuart equipment, Bibby Scientific, Stone, UK) for 2 h and 30 min. The mixture was centrifuged (Selecta JP Mixtasel, Abrera, Barcelona, Spain) for 10 min at 2000 rpm and the organic phase was collected in a new glass tube. The biomass residue was re-extracted twice with 2 mL of MeOH and 1 mL of CHCl_3_. To wash the lipid extract and induce phase separation, 2.3 mL of Milli-Q water was added to the final organic phase, followed by centrifugation for 10 min at 2000 rpm. The organic lower phase was collected in a new glass tube and dried under a nitrogen stream. Lipid extracts were then transferred to amber vials, dried again, weighed, and stored at −20 °C. Lipid content was estimated as a dry weight percentage. 

### 4.4. Fatty Acid Analysis by Gas Chromatography Mass Spectrometry

Fatty acid methyl esters (FAMEs) were prepared using a methanolic solution of potassium hydroxide (2.0 M) (Melo et al., 2015). A sample volume of 2 μL of hexane solution containing FAMEs was analyzed by gas chromatography mass spectrometry (GC-MS) on a GC system (Agilent Technologies 6890 N Network, Santa Clara, CA, USA) equipped with a DB-FFAP column with the following specifications: 30 m long, 0.32 mm internal diameter, and 0.25 μm film thickness (123–3232, J & W Scientific, Folsom, CA, USA). The GC equipment was connected to an Agilent 5973 Network Mass Selective Detector operating with an electron impact mode at 70 eV and scanning range *m/z* of 50 to 550 in a one second cycle in full scan mode acquisition. The oven temperature was programmed from an initial temperature of 80 °C for 3 min, a linear increase to 160 °C at 25 °C min^−1^, followed by linear increase at 2 °C min^−1^ to 210 °C, then at 30 °C min^−1^ to 250 °C, standing at 250 °C for 10 min. The injector and detector temperatures were 220 and 280 °C, respectively. Helium was used as the carrier gas at a flow rate of 1.4 mL min^−1^. FA identification was performed considering the retention times and MS spectra of FA standards (Supelco 37 Component Fame Mix, Sigma-Aldrich), and by MS spectrum comparison with chemical databases (Wiley 275 library and AOCS lipid library). FAMEs of five analytical replicates were injected. The relative amounts of FAs were calculated by the percent relative area method with proper normalization using internal standard methyl nonadecanoate (C19:0, Sigma-Aldrich, St. Louis, MO, USA), considering the sum of all relative areas of identified FAs.

### 4.5. Polar Lipid Analysis by Hydrophilic Interaction Liquid Chromatography Mass Spectrometry (HILIC-ESI-MS)

Lipid extracts were analyzed by mass spectrometry using hydrophilic interaction liquid chromatography (HILIC) on an Ultimate 3000 Dionex (Thermo Fisher Scientific, Bremen, Germany) with an autosampler coupled to a Q-Exactive hybrid quadrupole mass spectrometer (Thermo Fisher, Scientific, Bremen, Germany). The elution method was previously described [21,24,25] and applied with some modifications. The system is based on two mobile phases: Mobile phase A (25% water, 50% acetonitrile, and 25% methanol) and mobile phase B (60% acetonitrile and 40% methanol), both with 1 mM ammonium acetate. Firstly, 40% of mobile phase A was held isocratically for 8 min, followed by a linear increase to 60% of mobile phase A within 7 min, and maintained for 5 minutes. After that, conditions returned to the initial settings in 15 minutes (5 min to decrease to 40% of phase A and a re-equilibration period of 10 min prior to the next injection). A volume of 5 µL of each sample, containing 5 µg of lipid extract in CHCl_3_, 4 µL of phospholipid standards mix (dMPC-0.02 µg, dMPE-0.02 µg, LPC-0.02 µg, dPPI-0.08 µg, dMPG-0.012 µg, dMPS-0.04 µg, NPSM-0.02 µg, dMPA-0.08 µg, tMCL-0.08 µg), and 91 µL of starting eluent (60% B and 40% A), was introduced into the Ascentis Si column HPLC Pore column (15 cm × 1 mm, 3 µm, Sigma-Aldrich) with a flow rate of 40 µL min^−1^ at 30 °C. The mass spectrometer with Orbitrap^®^ technology was operated in simultaneous positive (electrospray voltage 3.0 kV) and negative (electrospray voltage ‒2.7 kV) modes with high resolution with 70,000 and AGC target of 1 × 10^6^, the capillary temperature was 250 °C, and the sheath gas flow was 15 U. The tandem mass spectrometry experiments were performed at a resolution of 17,500 and AGC target of 1 × 10^5^ with one full scan mass spectrum and 10 data-dependent MS/MS scans. The cycles were repeated continuously throughout the experiments with the dynamic exclusion of 60 s and an intensity threshold of 1 × 10^4^. Normalized collision energy™ (CE) ranged between 25, 30, and 35 eV. Data acquisition was performed using the Xcalibur data system (V3.3, Thermo Fisher Scientific, USA). The identification of molecular species of polar lipids was based on the assignment of the molecular ions observed in the LC-MS spectra, typical retention time, mass accuracy, and MS/MS spectra information. 

### 4.6. Data Analysis

In a first approach, the lipid species of *P. palmata* were identified using MZmine 2.32 software. This tool allows the analysis of raw data acquired in full MS by the integration of each lipid species peak, peak processing, and assignment against an in-house database. The validated peaks were within the time range of a MS full run and peaks with raw intensity lower than 1e4 were excluded. All the information originating from the MZmine software was confirmed based on the assignment of the molecular ions observed in the LC-MS spectra, typical retention time, exact mass accuracy, and MS/MS spectra information. Only exact mass accuracy with an error of less than 5 ppm was considered. The typical fragmentation of each lipid class in MS/MS spectra was taken into account according to what has already been described in the literature [25]. The normalization of the identified lipid species was performed by exporting integrated peak areas values (.csv file) and dividing the peak area value of each species by the peak area value of a standard lipid species with the closest retention time. The lipid species’ relative abundance was calculated in terms of the percentage dividing the normalized value of each lipid species by the sum of all identified lipid species. Bar graphs were created using the software GraphPad Prism 8.0.1

### 4.7. 2-Diphenyl-1-Picrylhydrazyl Radical Assay—DPPH Radical Scavenging Activity 

The antioxidant scavenging activity against the α,α-diphenyl-β-picrylhydrazyl radical (DPPH^●^) was evaluated using a previously described method [75,76] applied with some modifications. A stock solution of DPPH^●^ in ethanol (250 μmol/L) was prepared and diluted to provide a working solution with an absorbance value of ~0.9 measured at 517 nm using a UV-vis spectrophotometer (Multiskan GO 1.00.38, Thermo Scientific, Hudson, NH, USA) controlled by the SkanIT software version 3.2 (Thermo Scientific). To evaluate the radical stability, a volume of 150 μL of ethanol was added to 15 microplate wells followed by addition of 150 μL of DPPH^●^ diluted solution and an incubation period of 120 minutes, with absorbance measured at 517 nm every 5 minutes. For evaluation of the radical scavenging potential, a volume of 150 μL of *P. palmata* lipid extract (25, 50, 100, 250 μg/mL in ethanol) and 150 μL of Trolox standard solution (5, 12.5, 25, 37.5 μmol/L in ethanol) were placed in each well followed by addition of 150 μL of DPPH^●^ diluted solution, and again an incubation period of 120 minutes, with absorbance measured at 517 nm every 5 minutes. The control lipid extracts were also assayed by replacing 150 μL of DPPH^●^ diluted solution by 150 μL of ethanol. Radical reduction by hydrogen donor antioxidants was monitored by measuring the decrease in absorbance during the reaction, thereby quantifying radical scavenging, which is accompanied by a radical color change. All measurements were performed in triplicate on two different days. The % of DPPH radical remaining was determined according to:

*% DPPH remaining = (Abs samples after 120 min/Abs sample at the beginning of reaction) × 100*.

The free radical-scavenging activity of samples was determined as the percentage of inhibition of DPPH radical according to:

*Inhibition% = ((Abs DPPH – (Abs samples ‒ Abs control)) / Abs DPPH) × 100*.

The concentration of samples capable of reducing 30% of DPPH radical after 120 minutes (IC30) were calculated by linear regression using the concentration of samples and the percentage of the inhibition curve. The activity is expressed as Trolox Equivalents (TE, μmol Trolox/g of sample), according to:

*TE = IC30 Trolox (μmol/L) × 1000/IC30 of samples (μg/mL)*.

### 4.8. 2,20-Azino-bis-3-Ethylbenzothiazoline-6-Sulfonic Acid Radical Cation Assay—ABTS Radical Scavenging Activity 

The antioxidant scavenging activity against the 2,20-azino-bis-3-ethylbenzothiazoline-6-sulfonic acid radical cation (ABTS^●+^) was evaluated using a previously described method [76,77] applied with some modifications. The ABTS radical solution (3.5 mmol/L) was prepared by mixing 10 mL of ABTS stock solution (7 mmol/L in water) with 10 mL of potassium persulfate K_2_S_2_O_8_ (2.45 mmol/L in water) [77,78]. This mixture was kept for 12 h at room temperature and was diluted in ethanol to obtain an absorbance value of ~0.9 measured at 734 nm using a UV-vis spectrophotometer (Multiskan GO 1.00.38, Thermo Scientific, Hudson, NH, USA) controlled by the SkanIT software version 3.2 (Thermo Scientific). For an evaluation of the radical stability, a volume of 150 μL of ethanol was added to 15 microplate wells followed by addition of 150 μL of ABTS^●+^ diluted solution and an incubation period of 120 minutes, with absorbance measured at 734 nm every 5 minutes. For an evaluation of the radical scavenging potential, a volume of 150 μL of lipid extract (25, 50, 100, 250 μg/mL in ethanol) and 150 μL of Trolox standard solution (4, 8, 16, 28, 40, 56 μmol/L in ethanol) were placed in each well followed by addition of 150 μL of ABTS^●+^ diluted solution, and a new incubation period of 120 minutes, with absorbance measurements at 734 nm every 5 minutes. The control lipid extracts were also assayed by replacing 150 μL of ABTS^●+^ diluted solution by 150 μL of ethanol. Radical reduction by hydrogen donor antioxidants was monitored by measuring the decrease in absorbance during the reaction, thereby quantifying radical scavenging, which is accompanied by a radical color change. All measurements were performed in triplicate on two different days. The % of ABTS radical remaining was determined according to:

% *ABTS remaining = (Abs samples after 120 min/Abs sample at the beginning of reaction) × 100*.

The free radical-scavenging activity of samples was determined as the percentage of inhibition of ABTS radical according to:

*Inhibition% = ((Abs ABTS ‒ (Abs samples ‒ Abs control))/Abs ABTS) × 100*.

The concentration of samples capable of reducing 30% of ABTS radical after 120 minutes (IC50) were calculated by linear regression using the concentration of samples and the percentage of the inhibition curve. The activity is expressed as Trolox Equivalents (TE, μmol Trolox/g of sample), according to:

*TE = IC50 Trolox (μmol/L) × 1000/IC50 of samples (μg/mL)*.

## 5. Conclusions

In this study, the use of a mass spectrometry-based approach identified 144 lipid species of *P. palmata* produced in an environmentally friendly IMTA system. Eicosapentaenoic acid was the most abundant fatty acid identified in *P. palmata*, with a relative abundance greater than 50%. The content of EPA may be one of the major factors in the antioxidant activity of the polar lipid-rich extract, which has been found to be superior to that of phenolic compounds described in previous studies. Nevertheless, our results may not be exclusive to lipids, and synergy with other molecules should be taken into account. These findings revealed the lipid profile of *P. palmata* and highlighted that when farmed under IMTA, *P. palmata* can be a stable and sustainable source of beneficial lipids. The findings reported in this study add value to the polar lipid-rich extract of *P. palmata* and open a window of opportunity for innovative biotechnological applications targeting algal-based products. This work is the beginning of polar lipid prospection in *P. palmata* biomass and its results should now be validated across other possible *P. palmata* sources.

## Figures and Tables

**Table 1:** Fatty acid profile of *P. palmata* determined by GC-MS analysis. Abundances are expressed in relative abundance (%). Values are averages of five samples ± standard deviation. Double bond position of 18:1 and 18:2 was not identified.

Fatty Acid	Relative Abundance (%) ± SD
14:0	5.32 ± 0.44
16:0	24.32 ± 1.11
16:1(*n*-7)	2.03 ± 0.43
18:0	12.45 ± 6.74
18:1	2.82 ± 0.54
18:2	0.45 ± 0.19
20:4(*n*-6)	0.92 ± 0.17
20:5(*n*-3)	51.68 ± 6.47

**Table 2:** Molecular species of glycolipids SQDGs and SQMGs identified by HILIC-ESI-MS as [M − H]^−^ ions. C represents the total number of carbon atoms and N represents the total number of double bonds on the fatty acyl chains. The most abundant species in each class are highlighted in bold type. Fatty acids’ *sn*-1 and *sn*-2 position is based on biosynthetic pathways [27,28,29].

Theoretical *m/z*	Observed *m/z*	Error (ppm)	Lipid Species (C:N)	Fatty Acyl Chains	Formula
**527.2526**	**527.2541**	**2.84**	**SQMG(14:0)**	**14:0**	**C_23_H_43_O_11_S**
555.2839	555.2855	2.88	SQMG(16:0)	16:0	C_25_H_47_O_11_S
737.4510	737.4529	2.58	SQDG(28:0)	14:0/14:0	C_37_H_69_O_12_S
763.4666	763.4685	2.49	SQDG(30:1)	14:0/16:1	C_39_H_71_O_12_S
765.4823	765.4797	−3.40	SQDG(30:0)	14:0/16:0	C_39_H_73_O_12_S
785.4510	785.4531	2.67	SQDG(32:4)	16:3/16:1	C_41_H_69_O_12_S
787.4666	787.4630	−4.57	SQDG(32:3)	16:3/16:0	C_41_H_71_O_12_S
789.4823	789.4861	4.81	SQDG(32:2)	18:2/14:0	C_41_H_73_O_12_S
791.4979	791.5001	2.78	SQDG(32:1)	18:1/14:0	C_41_H_75_O_12_S
793.5136	793.5156	2.52	SQDG(32:0)	16:0/16:0	C_41_H_77_O_12_S
811.4666	811.4685	2.34	SQDG(34:5)	20:5/14:0	C_43_H_71_O_12_S
819.5292	819.5308	1.95	SQDG(34:1)	20:1/14:0	C_43_H_79_O_12_S
821.5449	821.5461	1.46	SQDG(34:0)	*	C_43_H_81_O_12_S
837.4823	837.4834	1.31	SQDG(36:6)	20:5/16:1	C_45_H_73_O_12_S
**839.4979**	**839.4998**	**2.26**	**SQDG(36:5)**	**20:5/16:0**	**C_45_H_75_O_12_S**
847.5605	847.5618	1.53	SQDG(36:1)	20:1/16:0	C_45_H_83_O_12_S
859.4666	859.4682	1.86	SQDG(38:9)	22:5/16:4 and 20:5/18:4	C_47_H_71_O_12_S
875.5918	875.5939	2.40	SQDG(38:1)	24:1/14:0	C_47_H_87_O_12_S
885.4823	885.4843	2.26	SQDG(40:10)	20:5/20:5	C_49_H_73_O_12_S

* No MS/MS information for FA composition.

**Table 3:** Molecular species of glycolipids MGDG, MGMG, DGDG, and DGMG identified by HILIC-ESI-MS as [M + NH4]^+^ ions. C represents the total number of carbon atoms and N represents the total number of double bonds on the fatty acyl chains. The most abundant species in each class are highlighted in bold type. Fatty acids’ *sn*-1 and *sn*-2 position is based on biosynthetic pathways [27,28,29].

Theoretical *m/z*	Observed *m/z*	Error (ppm)	Lipid Species (C:N)	Fatty Acyl Chains	Formula
482.3329	482.3326	−0.62	MGMG(14:0)	14:0	C_23_H_48_NO_9_
502.3016	502.3014	−0.40	MGMG(16:4)	16:4	C_25_H_44_NO_9_
504.3173	504.3184	2.18	MGMG(16:3)	16:3	C_25_H_46_NO_9_
506.3329	506.3331	0.39	MGMG(16:2)	16:2	C_25_H_48_NO_9_
510.3642	510.3640	−0.39	MGMG(16:0)	16:0	C_25_H_52_NO_9_
530.3329	530.3330	0.19	MGMG(18:4)	18:4	C_27_H_48_NO_9_
536.3799	536.3799	0.00	MGMG(18:1)	18:1	C_27_H_54_NO_9_
**556.3486**	**556.3485**	−**0.18**	**MGMG(20:5)**	**20:5**	**C_29_H_50_NO_9_**
766.5469	766.5494	3.26	MGDG(34:5)	20:5/14:0	C_43_H_76_NO^10^
774.6090	774.6104	1.81	MGDG(34:1)	18:1/16:0	C_43_H_84_NO_10_
794.5782	794.5778	−0.50	MGDG(36:5)	20:5/16:0	C_45_H_80_NO_10_
822.6095	822.6101	0.73	MGDG(38:5)	*	C_47_H_84_NO_10_
**840.5626**	**840.5627**	**0.12**	**MGDG(40:10)**	**20:5/20:5**	**C_49_H_78_NO_10_**
904.6878	904.6877	−0.11	MGDG(44:6)	*	C_53_H_94_NO_10_
**672.4170**	**672.4172**	**0.30**	**DGMG(16:0)**	**16:0**	**C_31_H_62_NO_14_**
698.4327	698.4326	−0.11	DGMG(18:1)	18:1	C_33_H_64_NO_14_
718.4014	718.4015	0.14	DGMG(20:5)	20:5	C_35_H_60_NO_14_
882.6154	882.6173	2.15	DGDG(30:0)	*	C_45_H_88_O_15_N
906.6154	906.6166	1.32	DGDG(32:2)	*	C_47_H_88_O_15_N
908.6310	908.6309	−0.11	DGDG(32:1)	18:1/14:0	C_47_H_90_O_15_N
928.5997	928.5997	0.00	DGDG(34:5)	20:5/14:0	C_49_H_86_O_15_N
932.6310	932.6319	0.97	DGDG(34:3)	*	C_49_H_90_O_15_N
934.6467	934.645	−1.82	DGDG(34:2)	*	C_49_H_92_O_15_N
936.6623	936.6623	0.00	DGDG(34:1)	18:1/16:0	C_49_H_94_O_15_N
**956.6310**	**956.6317**	**0.73**	**DGDG(36:5)**	**20:5/16:0**	**C_51_H_90_O_15_N**
984.6623	984.6617	−0.61	DGDG(38:5)	*	C_53_H_94_O_15_N
1002.6154	1002.6160	0.60	DGDG(40:10)	20:5/20:5	C_55_H_88_O_15_N

* No MS/MS information for FA composition.

**Table 4:** Molecular species of phospholipids LPC, PC, LPE, and PE identified by HILIC-ESI-MS as positive [M + H]^+^ ions. C represents the total number of carbon atoms and N represents the total number of double bonds on the fatty acyl chains. The most abundant species in each class are highlighted in bold type. Fatty acids’ *sn*-1 and *sn*-2 position is based on biosynthetic pathways [27,28,29].

Theoretical *m/z*	Observed *m/z*	Error (ppm)	Lipid Species (C:N)	Fatty Acyl Chains	Formula
468.3090	468.3092	0.43	LPC(14:0)	14:0	C_22_H_47_NO_7_P
494.3247	494.3242	−1.01	LPC(16:1)	16:1	C_24_H_49_NO_7_P
496.3403	496.3401	−0.40	LPC(16:0)	16:0	C_24_H_51_NO_7_P
516.3090	516.3090	0.00	LPC(18:4)	18:4	C_26_H_47_NO_7_P
518.3247	518.3236	−2.12	LPC(18:3)	18:3	C_26_H_49_NO_7_P
520.3403	520.3395	−1.54	LPC(18:2)	18:2	C_26_H_51_NO_7_P
522.3560	522.3564	0.77	LPC(18:1)	18:1	C_26_H_53_NO_7_P
**542.3247**	**542.3246**	−**0.18**	**LPC(20:5)**	**20:5**	**C_28_H_49_NO_7_P**
568.3403	568.3405	0.35	LPC(22:6)	22:6	C_30_H_51_NO_7_P
570.3560	570.3559	−0.18	LPC(22:5)	22:5	C_30_H_53_NO_7_P
706.5387	706.5387	0.00	PC(30:0)	14:0/16:0	C_38_H_77_NO_8_P
724.4917	724.4916	−0.14	PC(32:5)	*	C_40_H_71_NO_8_P
726.5074	726.5070	−0.55	PC(32:4)	14:0/18:4	C_40_H_73_NO_8_P
728.5230	728.5233	0.41	PC(32:3)	14:0/18:3	C_40_H_75_NO_8_P
730.5387	730.5387	0.00	PC(32:2)	14:0/18:2 and 16:0/16:2	C_40_H_77_NO_8_P
732.5543	732.5541	−0.27	PC(32:1)	16:0/16:1 and 14:0/18:1	C_40_H_79_NO_8_P
734.5700	734.5696	−0.54	PC(32:0)	*	C_40_H_81_NO_8_P
750.5074	750.5059	−2.00	PC(34:6)	*	C_42_H_73_NO_8_P
752.5230	752.523	0.00	PC(34:5)	14:0/20:5	C_42_H_75_NO_8_P
754.5387	754.538	−0.93	PC(34:4)	16:0/18:4	C_42_H_77_NO_8_P
756.5543	756.5541	−0.26	PC(34:3)	16:0/18:3	C_42_H_79_NO_8_P
758.5700	758.5699	−0.13	PC(34:2)	16:0/18:2	C_42_H_81_NO_8_P
760.5856	760.5856	0.00	PC(34:1)	16:0/18:1	C_42_H_83_NO_8_P
774.5073	774.5058	−1.94	PC(36:8)	16:3/20:5	C_44_H_73_NO_8_P
776.5230	776.5215	−1.93	PC(36:7)	16:2/20:5	C_44_H_75_NO_8_P
778.5387	778.5386	−0.13	PC(36:6)	16:1/20:5	C_44_H_77_NO_8_P
780.5543	780.5544	0.13	PC(36:5)	16:0/20:5	C_44_H_79_NO_8_P
784.5856	784.5823	−4.21	PC(36:3)	*	C_44_H_83_NO_8_P
786.6013	786.6002	−1.40	PC(36:2)	*	C_44_H_85_NO_8_P
788.6169	788.6153	−2.03	PC(36:1)	14:0/22:1	C_44_H_87_NO_8_P
800.5230	800.5225	−0.62	PC(38:9)	18:4/20:5	C_46_H_75_NO_8_P
802.5387	802.5362	−3.12	PC(38:8)	18:3/20:5	C_46_H_77_NO_8_P
806.5700	806.5699	−0.12	PC(38:6)	18:1/20:5	C_46_H_81_NO_8_P
808.5856	808.5845	−1.36	PC(38:5)	18:0/20:5	C_46_H_83_NO_8_P
810.6013	810.597	−5.30	PC(38:4)	*	C_46_H_85_NO_8_P
816.6482	816.6486	0.49	PC(38:1)	*	C_46_H_91_NO_8_P
**826.5387**	**826.5391**	**0.48**	**PC(40:10)**	**20:5/20:5**	**C_48_H_77_NO_8_P**
834.6013	834.5997	−1.92	PC(40:6)	20:1/20:5	C_48_H_85_NO_8_P
836.6169	836.6153	−1.91	PC(40:5)	*	C_48_H_87_NO_8_P
854.5700	854.5696	−0.47	PC(42:10)	22:5/20:5	C_50_H_81_NO_8_P
862.6326	862.6334	0.93	PC(42:6)	22:1/20:5	C_50_H_89_NO_8_P
864.6482	864.6459	−2.66	PC(42:5)	22:0/20:5	C_50_H_91_NO_8_P
882.6013	882.6026	1.47	PC(44:10)	*	C_52_H_85_NO_8_P
890.6639	890.6639	0.00	PC(44:6)	24:1/20:5	C_52_H_93_NO_8_P
480.3090	480.3092	0.42	LPE(18:1)	18:1	C_23_H_47_NO_7_P
**500.2777**	**500.2776**	−**0.20**	**LPE(20:5)**	**20:5**	**C_25_H_43_NO_7_P**
658.4448	658.4426	−3.34	PE(30:3)	*	C_35_H_65_NO_8_P
688.4917	688.4936	2.76	PE(32:2)	16:1/16:1	C_37_H_71_NO_8_P
690.5074	690.5088	2.03	PE(32:1)	16:0/16:1 and 14:0/18:1	C_37_H_73_NO_8_P
710.4761	710.4753	−1.13	PE(34:5)	14:0/20:5	C_39_H_69_NO_8_P
712.4917	712.4887	−4.21	PE(34:4)	16:0/18:4	C_39_H_71_NO_8_P
714.5074	714.5084	1.40	PE(34:3)	16:0/18:3 and 16:1/18:2	C_39_H_73_NO_8_P
716.5230	716.5233	0.42	PE(34:2)	16:1/18:1 and 16:0/18:2	C_39_H_75_NO_8_P
718.5387	718.5385	−0.28	PE(34:1)	16:0/18:1	C_39_H_77_NO_8_P
736.4917	736.4912	−0.68	PE(36:6)	*	C_41_H_71_NO_8_P
738.5074	738.5073	−0.14	PE(36:5)	16:0/20:5	C_41_H^73^NO_8_P
742.5387	742.5381	−0.81	PE(36:3)	*	C_41_H_77_NO_8_P
760.4917	760.489	−3.55	PE(38:8)	*	C_43_H_71_NO_8_P
764.5230	764.5239	1.18	PE(38:6)	18:1/20:5	C_43_H_75_NO_8_P
766.5387	766.5396	1.17	PE(38:5)	16:0/22:5 and 18:0/20:5	C_43_H_77_NO_8_P
**784.4917**	**784.492**	**0.38**	**PE(40:10)**	**20:5/20:5**	**C_45_H_71_NO_8_P**

* No MS/MS information for FA composition.

**Table 5:** Molecular species of phospholipids LPG, PG, PI, and PA identified by HILIC-ESI-MS as negative [M − H]^−^ ions. C represents the total number of carbon atoms and N represents the total number of double bonds on the fatty acyl chains. The most abundant species in each class are highlighted in bold type. Fatty acids’ *sn*-1 and *sn*-2 position is based on biosynthetic pathways [27,28,29].

Theoretical *m/z*	Observed *m/z*	Error (ppm)	Lipid Species (C:N)	Fatty Acyl Chains	Formula
**455.2410**	**455.2425**	**3.29**	**LPG(14:0)**	**14:0**	**C_20_H_40_O_9_P**
481.2566	481.258	2.91	LPG(16:1)	16:1	C_22_H_42_O_9_P
483.2723	483.2741	3.72	LPG(16:0)	16:0	C_22_H_44_O_9_P
529.2566	529.2583	3.21	LPG(20:5)	20:5	C_26_H_42_O_9_P
691.4550	691.4565	2.17	PG(30:1)	14:0/16:1	C_36_H_68_O_10_P
693.4707	693.4724	2.45	PG(30:0)	14:0/16:0	C_36_H_70_O_10_P
719.4863	719.4879	2.22	PG(32:1)	14:0/18:1 and 16:0/16:1	C_38_H_72_O_10_P
721.502	721.5039	2.63	PG(32:0)	16:0/16:0 and 14:0/18:0	C_38_H_74_O_10_P
739.455	739.4583	4.46	PG(34:5)	14:0/20:5	C_40_H_68_O_10_P
745.502	745.5033	1.74	PG(34:2)	16:0/18:2 and 16:1/18:1	C^40^H_74_O_10_P
747.5176	747.5189	1.74	PG(34:1)	16:0/18:1 and 14:0/20:1	C_40_H_76_O_10_P
749.5333	749.5345	1.60	PG(34:0)	16:0/18:0 and 14:0/20:0	C_40_H_78_O_10_P
**765.4707**	**765.4735**	**3.66**	**PG(36:6)**	**16:1/20:5**	**C_40_H_70_O_10_P**
767.4863	767.4871	1.04	PG(36:5)	*	C_42_H_72_O_10_P
773.5333	773.5348	1.94	PG(36:2)	18:1/18:1	C_42_H_78_O_10_P
775.5489	775.5508	2.45	PG(36:1)	14:0/22:1	C_42_H_80_O_10_P
777.5646	777.5662	2.06	PG(36:0)	*	C_42_H_82_O_10_P
783.4812	783.4810	−0.29	PG(36:5-OH)	16:0-OH/20:5	C_42_H_72_O_11_P
801.5646	801.5668	2.74	PG(38:2)	16:0/24:2	C_44_H_82_O_10_P
805.5959	805.5945	−1.74	PG(38:0)	*	C_44_H_86_O_10_P
813.4707	813.4736	3.60	PG(40:10)	20:5/20:5	C_46_H_70_O_10_P
829.5959	829.599	3.74	PG(40:2)	16:1/24:1 and 14:0/26:2	C_46_H_86_O_10_P
831.6115	831.6138	2.77	PG(40:1)	14:0/26:1	C_46_H_88_O_10_P
859.6428	859.6452	2.79	PG(42:1)	16:0/26:1	C_48_H_92_O_10_P
855.5024	855.5039	1.75	PI(36:5)	16:0/20:5	C_45_H_76_O_13_P
881.5180	881.5204	2.72	PI(38:6)	18:1/20:5	C_47_H_78_O_13_P
**901.4867**	**901.4888**	**2.33**	**PI(40:10)**	**20:5/20:5**	**C_49_H_74_O_13_P**
965.6119	965.6138	1.97	PI(44:6)	*	C_53_H_90_O_13_P
**739.4339**	**739.4346**	**0.95**	**PA(40:10)**	**20:5/20:5**	**C_43_H_64_O_8_P**
743.4652	743.4669	2.29	PA(40:8)	20:4/20:4	C_43_H_68_O_8_P

* No MS/MS information for FA composition.

**Table 6:** Molecular species of IPC identified by HILIC-ESI-MS as negative [M + CH_3_COO]^−^ ions. C represents the total number of carbon atoms and N represents the total number of double bonds on the fatty acyl chains. The most abundant species are highlighted in bold type.

Theoretical *m/z*	Observed *m/z*	Error (ppm)	Lipid Species (C:N)	Fatty Acyl Chains	Formula
892.5915	892.5933	2.02	PI-Cer(d38:2)	*	C_46_H_87_NO_13_P
918.6072	918.6096	2.61	PI-Cer(d40:3)	*	C_48_H_89_NO_13_P
**920.6228**	**920.6217**	**−1.19**	**PI-Cer(d40:2)**	*	**C_48_H_91_NO_13_P**
922.6385	922.6353	−3.47	PI-Cer(d40:1)	*	C_48_H_93_NO_13_P
946.6385	946.6403	1.90	PI-Cer(d42:3)	*	C_50_H_93_NO_13_P
948.6541	948.6535	−0.63	PI-Cer(d42:2)	*	C_50_H_95_NO_13_P

* No MS/MS information for FA composition.
